# Overexpression of Toll-like receptor 4 contributes to the internalization and elimination of *Escherichia coli* in sheep by enhancing caveolae-dependent endocytosis

**DOI:** 10.1186/s40104-021-00585-z

**Published:** 2021-05-10

**Authors:** Yao Li, Yue Zhao, Xueling Xu, Rui Zhang, Jinlong Zhang, Xiaosheng Zhang, Yan Li, Shoulong Deng, Zhengxing Lian

**Affiliations:** 1 Beijing Key Laboratory for Animal Genetic Improvement, National Engineering Laboratory for Animal Breeding, Key Laboratory of Animal Genetics and Breeding of the Ministry of Agriculture, College of Animal Science and Technology, China Agricultural University, Beijing, China; 2 Tianjin Institute of Animal Sciences, Tianjin, China; 3 State Key Laboratory of Agrobiotechnology, College of Biological Sciences, China Agricultural University, Beijing, China; 4 Institute of Laboratory Animal Sciences, Chinese Academy of Medical Sciences and Comparative Medicine Center, Peking Union Medical College, Beijing, China

**Keywords:** Caveolae-dependent endocytosis, Host defence infection, Inflammatory responses, Monocytes, Toll-like receptor 4

## Abstract

**Background:**

Gram-negative bacterial infections have a major economic impact on both the livestock industry and public health. Toll-like receptor 4 (TLR4) plays a crucial role in host defence against Gram-negative bacteria. Exploring the defence mechanism regulated by TLR4 may provide new targets for treatment of inflammation and control of bacterial infections. In a previous study, we generated transgenic sheep overexpressing *TLR4* by microinjection to improve disease resistance. The defence mechanism through which TLR4 overexpression protected these sheep against pathogens is still not fully understood.

**Results:**

In the present study, we used *Escherichia coli* to infect monocytes isolated from peripheral blood of the animal model. The overexpression of TLR4 strongly enhanced the percentage of endocytosis and capacity of elimination in monocytes during the early stages of infection. This phenomenon was mainly due to overexpression of TLR4 promoting caveolae-mediated endocytosis. Pretreatment of the transgenic sheep monocytes with inhibitors of TLR4, Src signalling, or the caveolae-mediated endocytosis pathway reduced the internalization of bacteria, weakened the ability of the monocytes to eliminate the bacteria, and increased the pH of the endosomes.

**Conclusion:**

Together, our results reveal the effects of TLR4 on the control of *E. coli* infection in the innate immunity of sheep and provide crucial evidence of the caveolae-mediated endocytosis pathway required for host resistance to invading bacteria in a large animal model, providing theoretical support for breeding disease resistance in the future. Furthermore, Src and caveolin 1 (CAV1) could be potentially valuable targets for the control of infectious diseases.

**Supplementary Information:**

The online version contains supplementary material available at 10.1186/s40104-021-00585-z.

## Background

Toll-like receptor 4 (TLR4) is known to be a pattern-recognition receptor that triggers innate immunity [1–3]. This molecule is expressed mainly in the immunocytes of mammals, including macrophages, monocytes, neutrophils, and dendritic cells [4]. As an important component of the body’s defence system, monocytes not only promote specific proinflammatory and anti-inflammatory processes but also endocytose and eliminate pathogens to maintain cell homeostasis [5]. TLR4 recognizes Gram-negative bacteria via their signature molecules to activate the host’s innate immune response, which is tightly regulated via distinct factors and pathways. The localization of TLR4 has emerged as a key determinant of TLR4 function. These factors include subcellular TLR4 localization and ligand sensing, which occurs on the cell surface through activation of the Toll-interleukin 1 receptor (TIR) domain containing adaptor protein (TIRAP)-myeloid differentiation factor 88 (MyD88)-dependent pathway and through endocytosis that activates the TRIF-related adaptor molecule (TRAM)-TIR domain containing adaptor-inducing interferon-β (TRIF)-dependent pathway [6]. TLR4 does not simultaneously activate the MyD88- and TRIF-dependent pathways; instead, it induces these two signalling pathways sequentially following the endocytosis of pathogens [7]. First, when bacteria bind to TLR4-bound myeloid differentiation protein 2 (MD2) on the plasma membrane, TIRAP and MyD88 participate in the rapid activation of the TIRAP-MyD88-dependent pathway, which results in the production of proinflammatory cytokines [8]. Next, TLR4 enters the endocytosis pathway by being translocated away from the cell surface to the endosome accompanied by cargo [9]. With the help of the bridging factor TRAM, TLR4 recruits the TRIF receptor that drives the TRAM-TRIF-dependent pathway and leads to the production of type I interferons [10].

The endocytosis of pathogens has emerged as a critical control step in the antimicrobial signal transduction process [11]. Macrophages with defects in the internalization of cell surface TLR4 exhibit enhanced inflammatory cytokine production following stimulation with lipopolysaccharide (LPS) and fail to induce LPS tolerance despite repeated LPS stimulation [12]. The internalization of TLR4 into the endosome is one of the negative regulatory mechanisms that prevent an excessive inflammatory response [13].

After immunocytes internalize invading pathogens by endocytosis, a series of vesicular trafficking steps occur with organelles ranging from early endosomes to lysosomes. At the end of this process, the cargo can be effectively degraded when mature endosomes fuse with endolysosomes [14]. Acidification is essential during endosome maturation: a sufficiently low pH is a prerequisite for endosome-lysosome fusion and provides optimal conditions for the activity of certain hydrolytic enzymes [15]. TLR4 is reported to control the rhythm of endosomal maturation and to guide bacteria into the “fast lane” of antigen presentation compared to apoptotic cell (not involved in TLR4) degradation by phagocytes. The inhibition of TLR4 downstream signalling events, such as mitogen-activated protein kinase (MAPK), blocks this enhanced endosomal maturation [16]. Research on *Salmonella* has found that TLR4 signalling enhances the acidification rate of endosomes containing *Salmonella* [17]. However, the mechanisms linking TLR4 and endosome maturation remain to be fully explored.

Endocytosis occurs via a variety of mechanisms in mammalian cells, including clathrin-dependent endocytosis, micropinocytosis, phagocytosis, and caveolae-dependent endocytosis [18, 19]. Caveolae are submicroscopic plasma membrane lipid rafts that are rich in cholesterol and sphingolipids and found in many mammalian cell types [20]. Caveolae have vital functions in signal transduction and the elimination of bacteria [21]. TLR4 activation has been associated with the translocation of TLR4 and signalling proteins into caveolae since the inhibition of the caveolae-dependent endocytosis pathway blocks LPS-induced TLR4 signalling [22]. Caveolin 1 (CAV1) is the principal structural and signalling component of caveolae and has been used as a marker protein [23]. This molecule has been shown to be involved in endocytosis. The findings from a mouse model of sepsis indicated that CAV1 is an important protective modulator of sepsis, as CAV1 may regulate inflammation and reduce the bacterial burden [24]. Studies on mouse macrophages found that knocking out *CAV1* reduces their capacity for endocytosis and their ability to kill bacteria. In addition, the expression of TLR4 and MyD88 was decreased, and the production of inflammatory cytokines was also decreased [25]. Several studies have found that CAV1 can be phosphorylated by the tyrosine kinase Src, and activated CAV1 promotes caveolae-mediated endocytosis [21, 26]. Furthermore, the Src kinase family phosphorylates TLR4 to dissociate MyD88 and MyD88 adapter-like (MAL)/TIRAP, thereby suppressing LPS-induced inflammatory responses [27]. These results indicate the potential role of TLR4 in caveolae-dependent endocytosis and signalling, although the precise mechanisms remain unknown.

In our previous study, we bred transgenic sheep overexpressing *TLR4* [28, 29]. These transgenic sheep produced a stronger inflammatory response during the early stages of bacterial infection than control sheep, and the infection subsided quickly. TLR4 is known to increase the expression of scavenger receptors, which enhance the adhesion capacity of immunocytes and contribute to the endocytosis of pathogenic bacteria [30, 31]. However, the effect of TLR4 overexpression on immune defence mechanisms has not been completely elucidated. Here, we continued this line of investigation by determining the effects of TLR4 on bacterial endocytosis and the bactericidal ability. We further examined whether the immunocytes of transgenic sheep have improved antibacterial ability via caveolae-dependent endocytosis.

## Methods

### Sheep

**Fig. 1:**
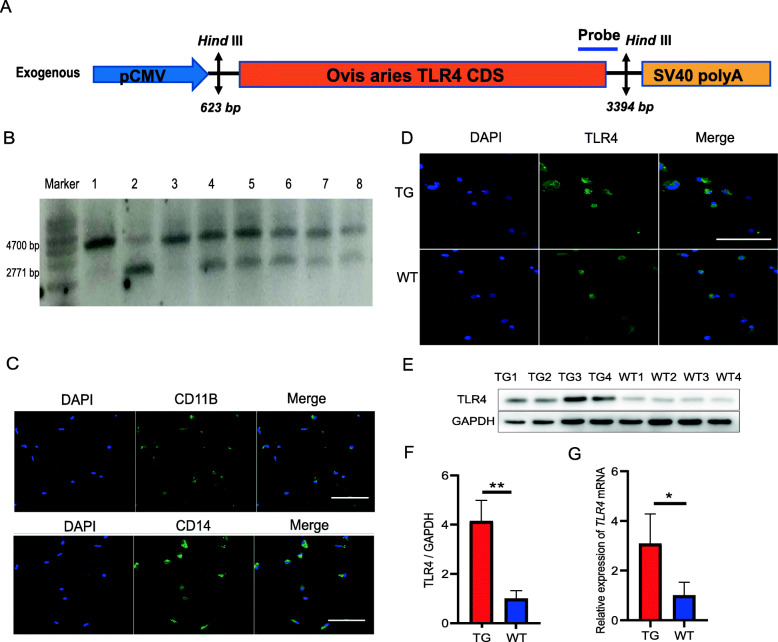
Detection of TLR4 overexpression in ovine blood monocytes. **a** Schematic diagram of the structure of the CMV-*Ovis aries TLR4* overexpression vector. **b** Representative Southern blot used to identify transgenic (TG) sheep based on the presence of the *TLR4* transgene. Partially TG sheep had both the endogenous 4700-bp *TLR4* band and the exogenous 2771-bp *TLR4* band. Marker, 1-kb ladder; lanes 1–8, eight individuals comprising the wild-type (WT) sheep in lanes 1 and 3 and the TG sheep in lanes 2 and 4–8. **c** Monocytes were isolated from four WT and four TG sheep and stained for the expression of the monocyte markers, CD14 and CD11b. DAPI was used to stain the cell nuclei. Scale bar: 100 μm. **d** Similarly, TLR4 expression in the TG and WT monocytes was examined by immunofluorescence microscopy. Scale bar: 100 μm. **e** The protein expression of TLR4 in monocytes was examined by western blotting. **f** Quantification of the data in (**e**). **g** The mRNA expression of *TLR4* in monocytes was evaluated by qRT-PCR. Both the mRNA and protein expression levels of TLR4 were significantly higher in the TG monocytes than in the WT monocytes. All data are presented as the mean ± SD, *n* ≥ 3; *P < 0.05, **P < 0.01. *GAPDH* was used for normalization

The founder transgenic sheep were produced through microinjection of a linear vector containing sheep *TLR4* (Fig. 1a) into the pronucleus of fertilized eggs [28, 29]. Founder transgenic sheep were bred with wild-type sheep. Among the offspring of the transgenic sheep, healthy male 2- to 3-year-old sheep were identified as either wild-type or transgenic by Southern blotting. Genomic DNA (20 μg) was extracted from the ear tissue and digested with *Hind*III (New England Biolabs, Ipswich, Britain). The probe used for Southern blotting was generated by PCR with the following primer pair: forward (F), 5′-ACTGGTAAAGAACTTGGAGGAGG-3′ and reverse (R), 5′-CCTTCACAGCATTCAACAGACC-3′, and the 671-bp PCR product was labelled with digoxigenin (Roche Diagnostics, Mannheim, Germany). Four sheep were included in the transgenic and four sheep in wild-type groups for the subsequent experiments.

### Cells and culture conditions

The initial peripheral blood mononuclear cells were isolated from the peripheral blood of sheep that had been collected aseptically from the jugular vein in separation medium (TBDscience, Tianjing, China), followed by density gradient centrifugation (2,000×*g* for 30 min). The cells were seeded at a density of 1 × 10^6^ cells per well in 6-well culture plates. After incubation at 37 °C in a 5% CO_2_ incubator for 2 h, the nonadherent cells were removed by washing the wells three times with phosphate-buffered saline (PBS). The adherent cells were cultured at 37 °C in a 5% CO_2_ incubator with RPMI-1640 medium (Gibco, Grand Island, NY, USA) containing 10% foetal bovine serum (FBS; Gibco). After a further 48 h of incubation and the removal of nonadherent cells, the adherent cells were mainly composed of monocytes.

### Immunofluorescence assay

First, the monocytes were washed with PBS and then digested with 0.25% trypsin-EDTA (Gibco, Grand Island, NY, USA) at 37 °C for 5–10 min. After termination of the digestion, the cell suspension was poured into a 15 mL centrifuge tube and washed twice with PBS, RPMI-1640 medium was added, and a cell counting plate was used for counting. Monocytes were seeded on 8-well glass chamber slides (Millipore, Massachusetts, USA) at a density of 1 × 10^5^ cells per well. After 12 h, the cells were washed three times with PBS before being fixed with 4% paraformaldehyde for 20 min, followed by blocking with Immunol Staining Blocking Buffer (Beyotime, Beijing, China) for 1 h. Diluted primary antibodies against TLR4 (1:500; Affinity Biosciences, OH, USA) and cluster of differentiation 14 (CD14) and CD11b (1:500; Bioss, Beijing, China) were incubated with the cells at 4 °C for 12 h and a fluorescein isothiocyanate (FITC)- labelled secondary antibody (1:1,000, Beyotime) was applied according to the manufacturer’s instructions. The cell nuclei were stained with 4′,6-diamidino-2-phenylindole (DAPI; Solarbio, Beijing, China) before placing the coverslip over the slides along with an anti-fluorescence quenching agent. Fluorescent staining was observed with a laser scanning confocal microscope (Nikon Instruments Inc., NY, USA).

### Western blotting

The cells were washed three times with PBS before the total protein was harvested with the Minute Total Protein Extraction Kit (Invent Biotechnologies, Eden Prairie, USA). We added phosphatase inhibitor complex I (1:100, Aidlab, China) to lysis buffer (denaturing cell lysis buffer) prior to use. The protein concentration of each extract was quantified using the BCA Protein Assay Kit (Beyotime) according to the manufacturer’s protocols. After the crude lysate had been resuspended in sodium dodecyl sulphate (SDS) buffer and incubated at 95 °C for 10 min, equal amounts of the protein samples (about 20–30 μg each) were loaded in separate lanes of a 12% gel for SDS–polyacrylamide gel electrophoresis. Following electrophoretic separation, the proteins were transferred to a polyvinylidene fluoride membrane (Millipore, Massachusetts, USA). The membranes were blocked with 5% milk powder for 2 h at room temperature. After incubation overnight with antibodies against TLR4 (AF7017, 1:500; Affinity Biosciences, OH, USA), Src (2109S, 1:1,000; Cell Signaling Technology, Inc., Boston, USA), p-Src (2101S, 1:1,000; Cell Signaling Technology), CAV1 (3238S, 1:1,000; Cell Signaling Technology), p-CAV1 (3251S, 1:1,000; Cell Signaling Technology), and GAPDH (D110016, 1:5,000; Sangon, Shanghai, China) at 4 °C, the membranes were washed thoroughly with Tris-buffered saline containing 0.1% Tween 20 (TBST). The membranes were then incubated with a horseradish peroxidase-conjugated secondary antibody (A0208, 1:5,000; Beyotime). The membranes were visualized with an enhanced chemiluminescence system (Solarbio) according to the manufacturer’s instructions.

### Monocyte infection and colony-forming unit (CFU) counts

The *E. coli* K12 strain DH5a and *Staphylococcus aureus* RN4220 were cultured in Luria-Bertani (LB) broth at 37 °C. The bacterial growth phase was measured by the absorbance of the bacterial suspension at 600 nm. An optical density (OD) of ~ 0.4 ensured that bacteria were in the logarithmic growth phase. The number of bacteria was counted by plate counting through serial 10-fold dilutions of the inoculum to LB agar. The bacteria were suspended and diluted to a concentration of 1 × 10^7^ cells/mL in RPMI-1640 medium (Thermo Fisher Scientific, Waltham, USA) containing 10% FBS. Before infection, monocytes were counted again and seeded in 6-well culture plates (Corning, NY, USA). The monocytes were cultured for 12 h to ensure cell adherence to the well bottoms, washed three times in PBS without antibiotics, mixed with bacteria at a multiplicity of infection (MOI) of 10, and then centrifuged at 200×*g* for 2 min. The monocytes were subsequently incubated at 37 °C in 5% CO_2_ for the indicated times. The cells were washed three times with PBS containing gentamicin (100 μg/mL) and lysed with 0.1% Triton X-100 (Sigma-Aldrich, Saint Louis, USA) to release intracellular bacteria. The total intracellular CFU of bacteria was quantified by culture in agar after serial dilution.

For determination of the bacterial killing capacity of the monocytes, all samples were first incubated with *E. coli* for the same period (15 min). The extracellular bacteria were removed by washing the samples three times with PBS containing gentamicin (100 μg/mL). RPMI-1640 supplemented with 10% FBS was added to the cells, and the cells were then incubated for the indicated times. The cells were washed twice with PBS and lysed with 0.1% Triton X-100. The time to phagocytosis was counted from the time of bacterial addition. The data on the numbers of killed bacteria were obtained by subtracting the number of intracellular bacteria at 30 min from the same number at 15 min.

### Quantitative reverse-transcription polymerase chain reaction (qRT-PCR)

The qRT-PCR reactions were performed on an MX3000P instrument (Agilent Technologies, Santa Clara, USA) with the SYBR Premix Ex Taq II kit (TaKaRa, Kyoto, Japan). The total RNA of the sheep peripheral blood monocytes was extracted with an RNA extraction kit (Aidlab, Beijing, China), and a PrimeScript RT kit (TaKaRa) was used to reverse transcribe the RNA into cDNA. Primer pairs were designed by the primer design software Primer 3 and confirmed by the Primer- BLAST tool at the NCBI (National Center of Biotechnology Information) site. A melting curve analysis confirmed the presence of a single gene specific peak and the absence of primer dimers. Melting curve analysis consisted of 95 °C for 15 s, 60 °C for 1 min, and 95 °C for 30 s.

The primer sequences used for qRT-PCR are presented in Supplementary Table S1.

For comparison of the expression of inflammatory factors between the transgenic and wild-type groups of sheep under *E. coli* challenge, the cells were treated with *E. coli* at a multiplicity of infection (MOI) of 10 and incubated for 30 min, and total RNA was extracted after first washing the cells three times with PBS. The mRNA expression levels were normalized to those of a housekeeping gene, *GAPDH*. The cells treated with *S. aureus* underwent the same procedure as the *E. coli* cells*.* The data were analysed using the comparative 2^−ΔΔCT^ method.

### Inhibitor treatment

For analysis of the interactions among TLR4, caveolae-dependent endocytosis, and Src signalling in *E. coli* internalization and elimination, monocytes were pretreated for 2 h with either 2 μmol/L Tak242 (MedChemExpress, Monmouth Junction, USA) —a TLR4 inhibitor, 3 μmol/L filipin—a caveolae-mediated endocytosis inhibitor (MedChemExpress), or 3 μmol/L dasatinib (MedChemExpress) —an Src inhibitor. After treatment, the cells were used for subsequent experiments.

### Monocyte endocytosis assay

*E. coli* were labelled with pHrodo from a pHrodo™ Red *E. coli* kit (Thermo Fisher Scientific). Before the powder was diluted in PBS (pH 7.4) to form a working solution, the mixture was sonicated for 30 min in the dark. Monocytes were plated in a 6-well plate (Corning) at 1× 10^6^ cells/well and incubated for 2 h at 37 °C. The monocytes were then infected with pHrodo- labelled *E. coli* at a ratio of 10:1 (bacteria:cells) in RPMI-1640 medium supplemented with 10% FBS. After centrifugation at 200×*g* for 2 min, the cells in the 6-well culture plates were further cultured for the indicated times. The assay was terminated at each time point by washing the cells three times with cold PBS, followed by the addition of 4% paraformaldehyde to each well. Subsequently, the cells were subjected to flow cytometry and microscopy studies.

### Flow cytometry

The percentage of monocyte endocytosis was measured by the uptake of red fluorescent pHrodo *Escherichia coli* (Thermo Fisher Scientific). Briefly, 1× 10^6^ cells/well monocytes were treated with pHrodo- labelled *E. coli* at a ratio of 10:1 (bacteria:cells) in a 6-well plate. After indicated times incubation, cells were washed and fixed with 4% paraformaldehyde then harvested in PBS pH 7.4. Samples were analysed by a flow cytometer (Beckman Coulter). Live cells were gated by forward scatter and side scatter area, singlets were gated by forward scatter area and forward scatter height. 1 × 10^4^ cells were collected, the PE channel was used to detect pHrodo-labeled *E. coli* in cells. The pHrodo threshold was set based on the sample of cells without pHrodo addition and applied to all samples. The percentage of monocyte endocytosis was the proportion of positive pHrodo cells in total cells. Dates were analysed with FlowJo software.

### Endosome pH

Each sample had the same monocytes in the test wells and standard curve wells. Monocytes were infected with pHrodo- labelled *E. coli* for 10 min in 96-well culture plates and then washed with cold PBS. Noninternalized pHrodo- labelled *E. coli* was washed away. In addition, this time was treated as the starting point (0 min). In standard curve wells, monocytes were treated for 30 min at a fixed pH (4.21, 5.18, 6.2, 7.38, or 8.16) with PBS containing 1% Triton X-100. Monocytes in the test wells tested at a predetermined time. The time when noninternalized pHrodo- labelled *E. coli* was washed away was taken as time 0. Fluorescence values were read at intervals using an emission wavelength of 590 nm. The cell luminescence value without pHrodo- labelled *E. coli* was used as the background control. The sample values were compared with standard curves obtained using each sample.

### Colocalization assay

*E. coli* was heat-inactivated at 90 °C for 30 min. After being washed twice with 0.1 mol/L NaHCO_3_ (pH 9.0), heat-inactivated *E. coli* was incubated in 0.1 mol/L NaHCO_3_ containing 1 mg/mL FITC (Sigma-Aldrich, Saint Louis, USA) for 1 h at room temperature with gentle stirring. Monocytes were plated onto 8-well glass chamber slides at a concentration of 1 × 10^5^ cells/well and incubated for 2 h at 37 °C to allow the cells to adhere. The cells were infected with FITC- labelled *E. coli* at an MOI of 10 for 30 min. Before incubation at 37 °C, the plates were centrifuged to synchronize endocytosis. The cells were washed three times with PBS after incubation and then fixed with 4% paraformaldehyde for 20 min, followed by the addition of 1% Triton X-100 to make the cells permeable. The cells were immunostained with EEA1 (ab2900, 1:500; Abcam, Cambridge, UK) or LAMP1 (ab67283, 1:500; Abcam). Alexa Fluor 647-conjugated anti­rabbit antibody (Beyotime) was used for detection.

### Cathepsin B activity assay

Monocytes were collected by centrifugation and lysed in 100 μL of prechilled cell lysis without inhibitors (Beyotime) on ice for 10 min. Then, the cells were centrifuged at top speed for 5 min, and the supernatant was transferred to a new tube. The protein concentration of each extract was quantified by a BCA Protein Assay Kit (Beyotime). The amount of total protein used for detection was the same. Each sample was measured using a cathepsin B activity assay kit (Yuanmu, Shanghai, China) according to the manufacturer’s protocols. The OD was determined at 450 nm. The sample value was obtained by the standard curve.

### Statistical analysis

All data are shown as the mean ± SD from no fewer than three independent experiments. The differences observed among samples were analysed with the independent-samples t-test and were considered statistically significant when *P* < 0.05.

## Results

### Detection of transgenic sheep overexpressing TLR4

The *TLR4* expression vector (Fig. 1a) was integrated into the genome of transgenic sheep bred during our previous study. Positive individuals were confirmed for use in the present study by Southern blotting. After digestion of the sheep genome with the restriction enzyme *Hind*III, the endogenous *TLR4* fragment (4700 bp) was found in all sheep, while the exogenous *TLR4* gene (2771 bp) was only identified in the transgenic sheep (Fig. 1b).

Monocytes are vital innate immune cells that play critical roles in the response to infection and inflammation through antigen presentation [32, 33]. Therefore, we isolated monocytes from ovine peripheral blood to determine the level of TLR4 expression in these cells. The monocyte markers CD14 and CD11b were detected on the cell membranes by antibody staining (Fig. 1c), and *TLR4* expression in the transgenic and wild-type sheep was compared at the transcription and protein levels (Fig. 1d–f). The results showed that the mRNA and protein levels of TLR4 were significantly higher (*P* < 0.05 and *P* < 0.01, respectively) in the transgenic ovine monocytes than in the wild-type monocytes.

### TLR4 overexpression leads to increased internalization and killing of *E. coli* and promotes the activation of multiple TLR4-mediated pathways

**Fig. 2:**
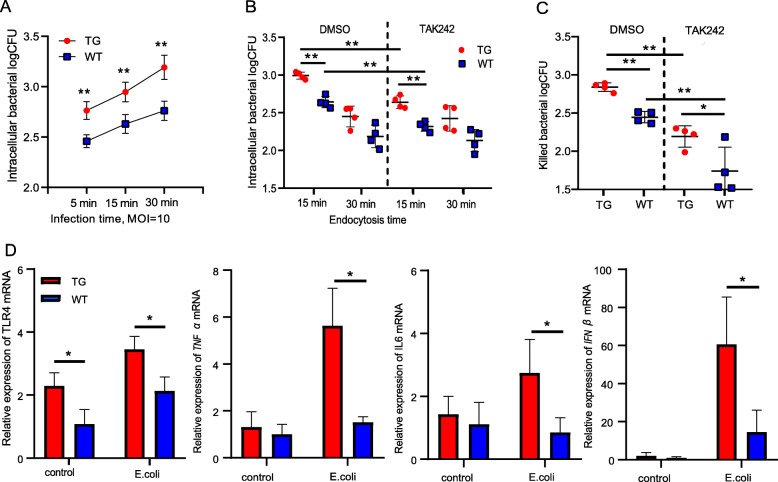
TLR4 overexpression promotes the activation of multiple pathways and increased *E. coli* internalization and killing. **a** Different infection times with *E. coli* (MOI = 10) and bacterial internalization was detected in transgenic (TG) and wild-type (WT) monocytes. **b** TG and WT monocytes were incubated with *E. coli* (MOI = 10) at the same time (15 min), the bacteria that were not internalized were washed away, and the monocytes were cultured further in the incubator. Bacterial levels in the monocytes were determined with a colony-forming unit (CFU) count, and the start of endocytosis (0 min) was when the bacteria were first added. The effect of the TLR4 inhibitor, TAK242, was also tested. **c** The numbers of bacteria killed in (**b**) were calculated by subtracting the number of intracellular bacteria at 30 min from the same number at 15 min. **d** The mRNA expression levels of *TLR4*, the proinflammatory cytokines *TNFα* and *IL-6*, and the anti-inflammatory cytokine *IFN-β* were determined in monocytes by qRT-PCR before and after *E. coli* infection for 30 min. All data are presented as the mean ± SD, *n* ≥ 3; *P < 0.05, **P < 0.01. DMSO, dimethyl sulfoxide (control); MOI, multiplicity of infection

The monocyte-mediated internalization of bacteria is an important antimicrobial reaction [34]. Here, the number of internalized *E. coli* in the TG and WT monocytes both increased with increasing infection time, although the levels of intracellular bacteria were significantly higher (*P* < 0.01 for all time points) in the TG monocytes than in the WT monocytes when using an MOI of 10 (Fig. 2a). However, the numbers of internalized *S. aureus* in the TG and WT monocytes were not different at any infection time (Fig. S1A). Next, the monocytes were challenged for a fixed time with *E. coli* and then cultured for the indicated times (Fig. 2b). The bacterial burden in the TG monocytes was significantly higher (*P* < 0.01) than that in the WT monocytes when the endocytosis time was 15 min. Both groups were treated with a TLR4 inhibitor, TAK242, which yielded similar results between the two groups (*P* < 0.01), in addition to a significant reduction in the number of bacteria carried by either group (*P* < 0.01). However, these differences between the TG and WT groups were not statistically significant after an endocytosis time of 30 min. The changing number of *E. coli* bacteria internalized by the cells over different endocytosis times can be attributed to the increased level of bacterial elimination by the monocytes with increasing endocytosis time (Fig. 2c). The elimination capacity of the TG group was significantly higher than that of the WT group regardless of whether the monocytes were treated with TAK242 (*P* < 0.01 without inhibition, *P* < 0.05 with inhibition). Furthermore, TAK242 significantly affected the bacterial killing capacity of both groups (*P* < 0.01).

The bacteria-induced activation of TLR4 often leads to the production of multiple cytokines to protect against infection [35, 36]. To elucidate how the overexpression of *TLR4* influences pathway activation, we examined the production of *TNFα* (tumour necrosis factor α) and *IL-6* (interleukin 6) by the TIRAP-MyD88-dependent pathway and the production of *IFN-β* (interferon β) by the TRAM-TRIF-dependent pathway by qRT-PCR during the early stages of infection. All cytokines were produced at significantly higher levels (*P* < 0.05) in the TG group than the WT group after 30 min of *E. coli* infection (Fig. 2d). In the *S. aureus* infection experiment, *TLR4* expression in the WT and TG groups did not change significantly after 30 min of incubation (MOI = 10) (Fig. S1B).

### TLR4 overexpression promotes monocyte endocytosis

**Fig. 3:**
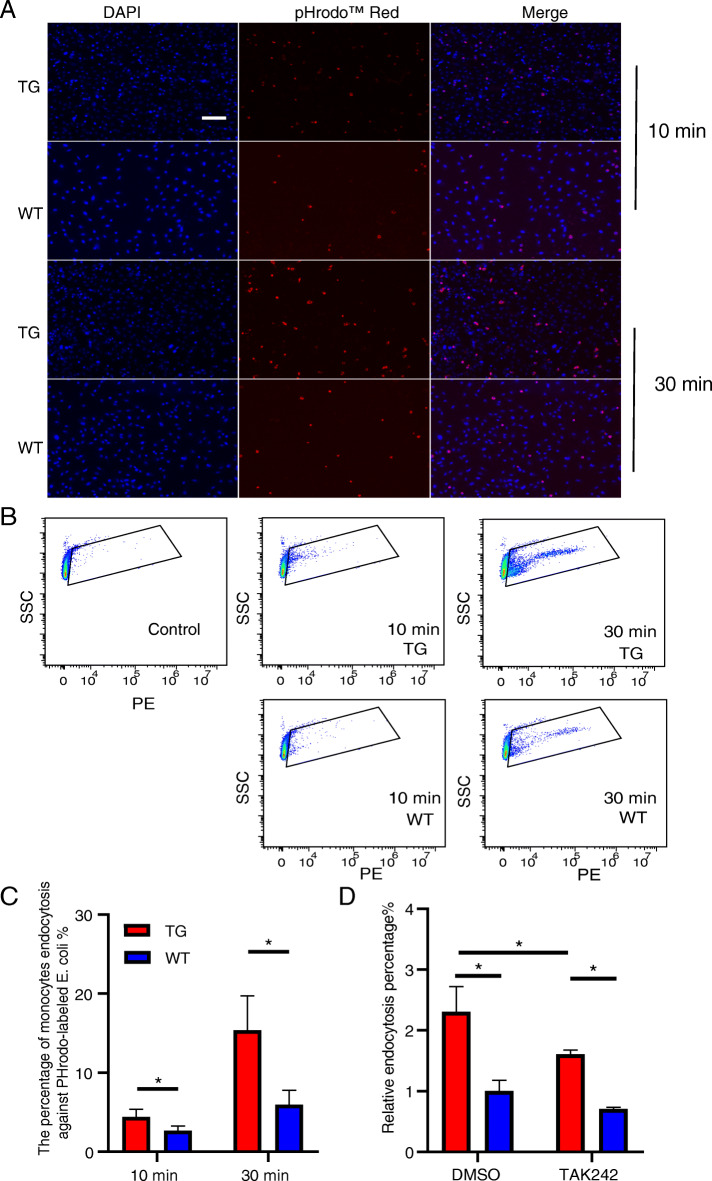
TLR4 overexpression promotes monocyte endocytosis. **a** The endocytosis of pHrodo-labelled *E. coli* by monocytes in the transgenic (TG) and wild-type (WT) groups was observed by fluorescence microscopy. Scale bar: 100 μm. **b** Flow cytometric analysis of the percentage of monocyte endocytosis of the pHrodo- labelled *E. coli* after 10 min and 30 min of infection. **c** The effect of pretreatment with the TLR4 inhibitor TAK242 on monocyte endocytosis of pHrodo- labelled *E. coli*. All data are presented as the mean ± SD, *n* ≥ 3; *P < 0.05, **P < 0.01. DMSO, dimethyl sulfoxide (control)

To explore the effect of TLR4 overexpression on the ability of monocytes to endocytose bacteria, we used *E. coli* labelled with pHrodo. This unique fluorogenic dye is only incorporated into acidic cellular compartments and allows detection of these organelles by increasing the fluorescence intensity as the pH of the surroundings becomes more acidic. Endocytosis is a process accompanied by a rapid decrease in endosomal pH [15]. Internalized bacteria labelled with pHrodo are fluorescent and easily observed by microscopy, while extracellular bacteria are not fluorescent in the medium and are therefore not visible [37]. The results showed that the overexpression of TLR4 during the initial stages of bacterial infection (10 and 30 min) led to more monocytes undergoing internalization, and the fluorescence signals from these cells were more intense (Fig. 3a). Flow cytometric analysis showed that the percentage of monocyte endocytosis of pHrodo- labelled *E. coli* in the TG group was significantly higher (*P* < 0.05) than that in the WT group for both *E. coli* infection times (Fig. 3b, c and Supplementary Fig. S2). After pretreatment with the TLR4 inhibitor, TAK242, this percentage was still significantly higher (*P* < 0.05) in the TG group than in the WT group, and the inhibitory effect of TAK242 on the TG monocytes was stronger than that on the WT monocytes (Fig. 3d and Supplementary Fig. S3). These results suggest that the overexpression of TLR4 enhances monocyte endocytosis of *E. coli*.

### TLR4 overexpression promotes caveolae formation

**Fig. 4:**
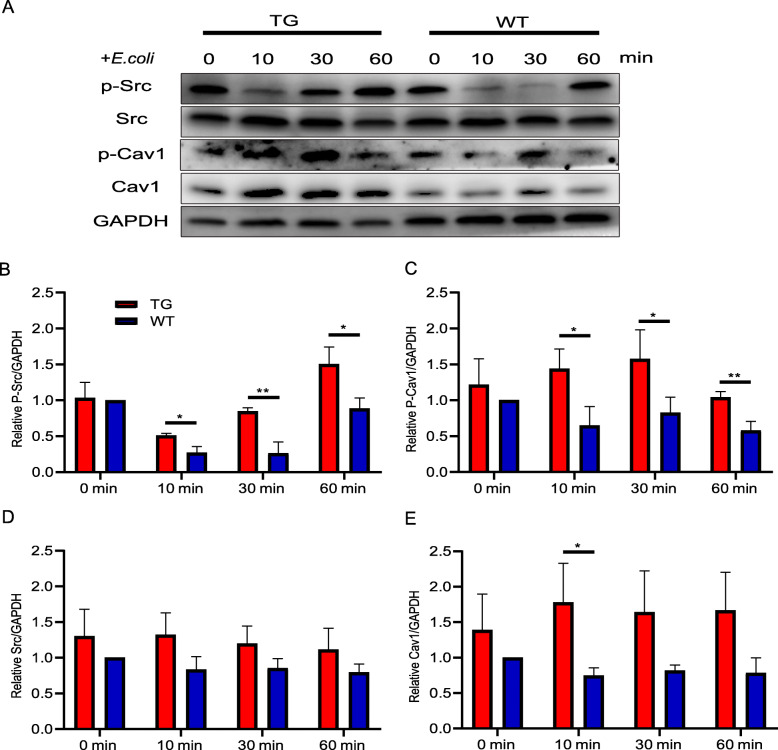
TLR4 overexpression promotes the internalization of *E. coli* through caveolae-mediated endocytosis. **a** The activation of proteins involved in caveolae-mediated endocytosis signalling in transgenic (TG) and wild-type (WT) monocytes was detected by immunoblotting at different time points after *E. coli* infection. **b**, **c** The phosphorylation levels of Src and CAV1 were quantified based on immunoblotting. **d**, **e** The protein expression levels of Src and CAV1 were quantified based on immunoblotting. All data are presented as the mean ± SD, *n* ≥ 3; *P < 0.05, **P < 0.01. GAPDH was used for normalization

Caveolae-mediated endocytosis is reported to be an extremely important pathway for realizing pathogen internalization [38], particularly towards *E. col* [39]. CAV1 is a signalling protein associated with caveolae; its phosphorylation by the tyrosine kinase Src activates CAV1 and promotes caveolae formation [20, 26]. To investigate the role of caveolae-mediated endocytosis in the underlying mechanism of TLR4 overexpression promoting *E. coli* endocytosis, the key proteins involved in caveolae formation, Src and CAV1, as well as their phosphorylation levels, were detected during the early stages of *E. coli* infection. The results showed that the phosphorylation of Src and CAV1 in the TG group started earlier than that in the WT group (Fig. 4a). Furthermore, at each time point, the levels of Src and CAV1 phosphorylation were significantly higher than those of the WT group. There were particularly significant differences in Src phosphorylation at 30 min and CAV1 phosphorylation at 60 min (*P* < 0.01 for both; Fig. 4b, c). However, the protein levels of Src and CAV1 were not significantly different between the two groups except for CAV1 at 10 min (Fig. 4d, e). These results suggest that the overexpression of TLR4 enhances Src and CAV1 phosphorylation, which can lead to increased levels of caveolae-mediated endocytosis during the initial stage of *E. coli* infection.

### Inhibition of caveolae-mediated endocytosis reduces the ability of TLR4-overexpressing monocytes to internalize and kill *E. coli*

**Fig. 5:**
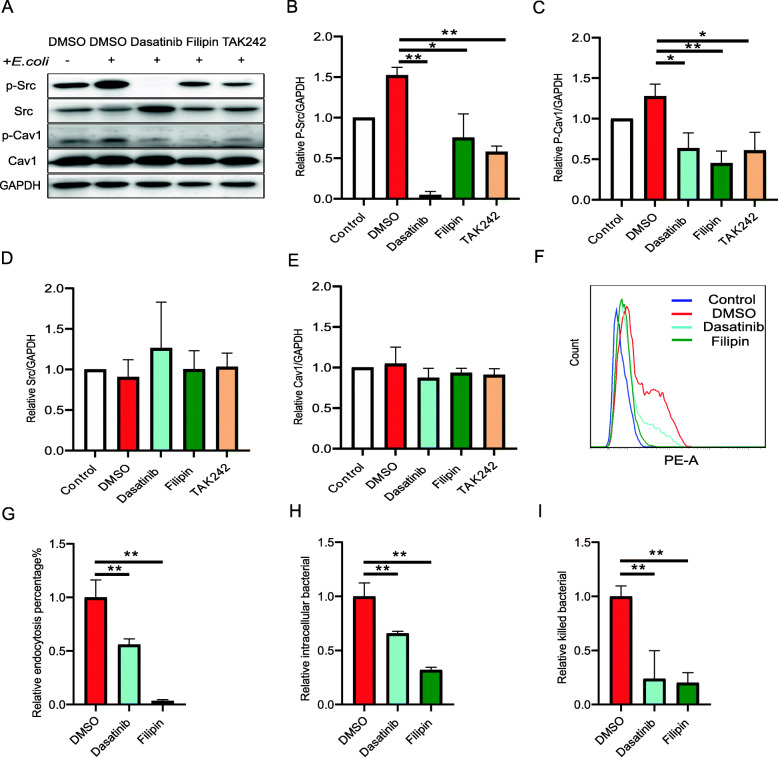
Inhibition of caveolae-mediated endocytosis reduces TLR4-overexpressing monocyte endocytosis and kills *E. coli*. **a** Monocytes were pretreated with the control (DMSO) or various inhibitors for 2 h, and after 30 min of *E. coli* infection (MOI = 10), the protein levels of Src, CAV1, p-Src, and p-CAV1 were determined by western blotting. **b**–**e** Quantification of the data in (**a**). GAPDH protein was used for normalization. **f**–**g** Flow cytometric analysis of the percentage of monocyte endocytosis of pHrodo- labelled *E. coli* (MOI = 10) by the TLR4-overexpressing cells pretreated with DMSO or the inhibitors dasatinib (3 μmol/L) or filipin (3 μmol/L) prior to 30 min of infection with pHrodo- labelled *E. coli*. **h** The quantity of *E. coli* internalized within monocytes after 30 min of infection (MOI = 10) was determined based on a colony-forming unit (CFU) count. **i** After 15 min of incubation with *E. coli* (MOI = 10), the bacteria that were not internalized were washed away, and the cells were cultured for an additional 15 min in the incubator. The number of *E. coli* killed in the monocytes was calculated based on the difference in the CFU values at these two time points. All data are presented as the mean ± SD, *n* ≥ 3; *P < 0.05, **P < 0.01. DMSO, dimethyl sulfoxide; MOI, multiplicity of infection

To better clarify the role of caveolae-mediated endocytosis in the antimicrobial activity of monocytes overexpressing TLR4, we performed experiments with various inhibitors. Prior to western blotting, TLR4-overexpressing monocytes were pretreated with either dasatinib (an Src inhibitor), filipin (a strong caveolae-mediated endocytosis inhibitor), or TAK242 (a TLR4 inhibitor) and subjected to 30 min of *E. coli* infection. The levels of phosphorylated Src and CAV1 in all treatment groups decreased (Fig. 5a–c), especially those of p-Src in the dasatinib and TAK242 treatment groups (*P* < 0.01) and p-CAV1 in the filipin treatment group (*P* < 0.01). However, there were no significant differences in the Src and CAV1 protein levels following inhibitor treatment (Fig. 5a, d, e). Flow cytometric analysis of the percentage of monocyte endocytosis of pHrodo- labelled *E. coli* in the untreated TLR4-overexpressing monocytes was compared with that of the dasatinib- and filipin-treated cells. Both inhibitors significantly reduced (*P* < 0.01) the percentage of endocytosis (Fig. 5f, g and Supplementary Fig. S4). The same results were observed for the intracellular *E. coli* CFU count (Fig. 5h) and the numbers of killed *E. coli* based on the CFU count (Fig. 5i). These results showed that the antimicrobial activity of the TLR4-overexpressing monocytes can be reduced by inhibitors of caveolae-mediated endocytosis. This finding suggests that caveolae-mediated endocytosis plays an important role in the antibacterial activity of monocytes overexpressing TLR4.

### TLR4 overexpression promotes endosome acidification and maturation

**Fig. 6:**
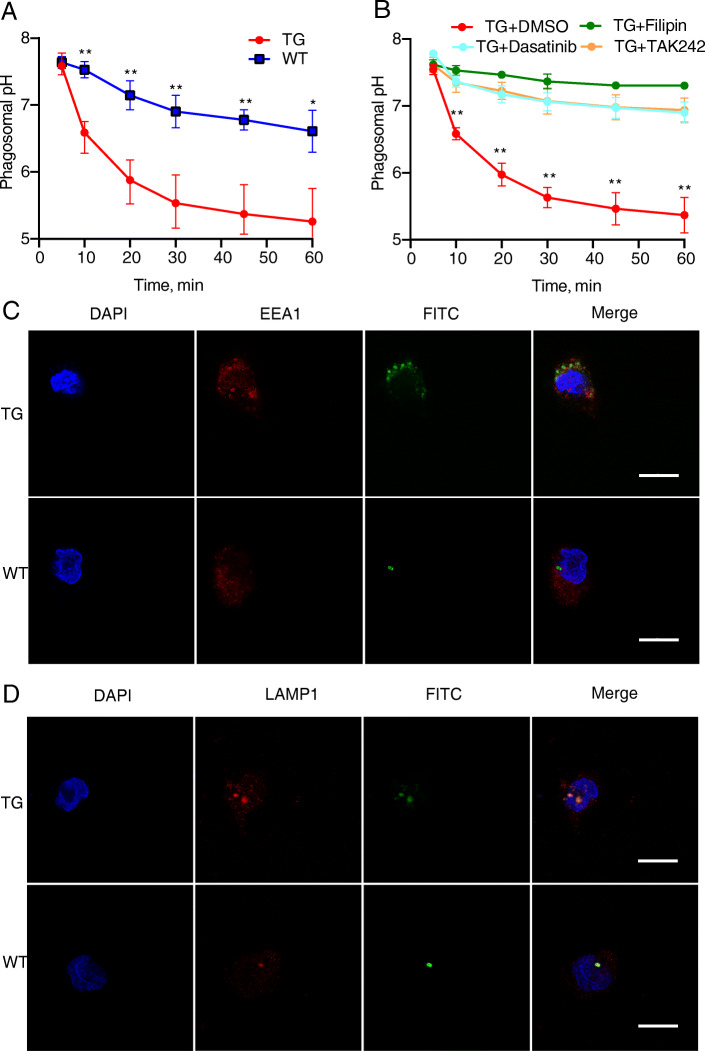
TLR4 overexpression promotes endosome maturation. **a** The endosomal pH values of transgenic (TG) and wild-type (WT) monocytes were assessed following infection with pHrodo- labelled *E. coli* (MOI = 10). Fluorescence values were read at intervals using an emission wavelength of 590 nm. Each sample had its own standard curves. The values were compared with standard curves obtained using corresponding sheep monocytes treated for 30 min at a fixed pH (4.21, 5.18, 6.2, 7.38, or 8.16) in phosphate-buffered saline containing 1% Triton X-100. The start of the experiment (0 min) was when the bacteria were first added. **b** Effects of inhibitor treatment (2 μmol/L TAK242, 3 μmol/L dasatinib, and 3 μmol/L filipin) on the endosomal pH of the TG and WT monocytes. Dimethyl sulfoxide (DMSO) was used as the control. **c** Fluorescence images of FITC- labelled *E. coli* colocalization with the primary endosomal marker EEA1 in the TG and WT monocytes after 30 min of infection. Scale bar: 10 μm. **d** Fluorescence images of FITC- labelled *E. coli* colocalization with the lysosomal marker LAMP1 in the TG and WT monocytes after 30 min of infection. Scale bar:10 μm. All data are presented as the mean ± SD, *n* ≥ 3; *P < 0.05, **P < 0.01. DAPI, cell nuclei stain

To elucidate the enhanced *E. coli* killing capacity of TG monocytes, we examined this issue from the perspective of endosome maturation. The function of the endosome network is to collect internalized cargoes and ferry them to their final destinations [40]. In this process, endosomes undergo a multitude of changes, including a drop in luminal pH. Here, pHrodo- labelled *E. coli* was used to compare endosomal pH changes between the TG and WT groups. Within 1 h after infection, the endosomal pH of the TG group exhibited a steeper decline than that of the WT group (Fig. 6a). Furthermore, the pH of the TG group was significantly lower than that of the WT group at all time points after 0 min (*P* < 0.05 to *P* < 0.01). When the TG cells were pretreated with the inhibitors dasatinib, filipin, and TAK242, all inhibitors were found to significantly reduce the acidification of the endosomes (*P* < 0.01 for all inhibitors and time points after 0 min; Fig. 6b). To further verify the influence of TLR4 overexpression on the maturation of the endosome, we used FITC- labelled *E. coli* to analyse colocalization with early endosome antigen 1 (EEA1, a primary endosomal marker) or lysosomal associated membrane protein 1 (LAMP1, a lysosomal marker). From the fluorescence images, it was observed that for both the EEA1 and LAMP1 markers, the TG group showed greater colocalization than the WT group (Fig. 6c, d). These results demonstrated that the overexpression of TLR4 can promote endosome acidification and that this acidification is regulated by the Src, caveolae-mediated endocytosis, and TLR4 pathways. In addition, the colocalization experiment with bacteria and endosomal markers for different stages of maturation provides another perspective on how the overexpression of TLR4 promotes endosomal maturation.

### TLR4 overexpression promotes cathepsin B activity

**Fig. 7:**
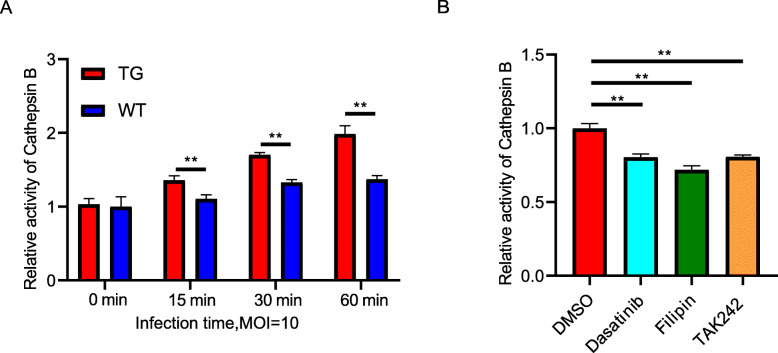
TLR4 overexpression enhances cathepsin B activity. **a** The relative cathepsin B activity of transgenic (TG) and wild-type (WT) monocytes was assessed following infection with *E. coli* for different times (MOI = 10). **b** Monocytes were pretreated with the control (DMSO) or various inhibitors for 2 h, and after 30 min of *E. coli* infection (MOI = 10), cathepsin B activity was compared. All data are presented as the mean ± SD, *n* ≥ 3; *P < 0.05, **P < 0.01. DMSO, dimethyl sulfoxide; MOI, multiplicity of infection

Cathepsin B is a kind of pH-activated protease that is important in protein degradation [41]. This molecule is present in endosomes, and changes in endosomal pH modulate cathepsin B activity [42]. Cathepsin B activity was examined to further clarify the effect of TLR4 overexpression on the protein degradation of monocytes. In our results, the cathepsin B activity in the TG monocytes was significantly higher (*P* < 0.01 for all time points except 0 min) than that in the WT monocytes when *E. coli* was infected using an MOI of 10. The levels in the TG group increased steeply with increasing infection time in an hour (Fig. 7a). After 30 min of infection with *E. coli*, the TG cells pretreated with the inhibitors dasatinib, filipin, and TAK242 had significantly reduced cathepsin B activity (*P* < 0.01) (Fig. 7b).

## Discussion

TLR4 is a pattern-recognition receptor that plays a key role in the resistance of host immune cells to Gram-negative bacteria. When TLR4 binds to its specific ligands, it triggers the activation of the innate immune response [43]. Several studies have focused on the function of TLR4 in bacterial infection. TLR4-deficient mice are more susceptible to infection than control mice and have higher bacterial loads [17, 44]. *TLR4*-mutant enterocytes exhibit decreased sensitivity to LPS compared with wild-type cells [45]. Mice overexpressing *TLR4* show greater protection against *Salmonella* infection because of their improved control of bacterial growth in target organs and the increased expression of important immune response genes in their spleen [46]. These results demonstrated that TLR4 has extraordinary functional importance in endocytosis and inflammatory regulation as part of the host defence. Mechanistic studies have shown that the overexpression of TLR4 can efficiently and rapidly activate the TIRAP-MyD88-dependent pathway, while also promoting the innate antiviral effect by activating the TRAM-TRIF-dependent pathway [30]. In addition, during the internalization of *E. coli*, the expression levels of TLR4, MAPKs, and scavenger receptors were all higher in the *TLR4*-overexpressing transgenic sheep than in the wild-type sheep. The level of actin polymerization and the adhesive capacity of sheep monocytes are also higher in the transgenic sheep than the control sheep [31]. However, the defence mechanisms involved in the protective effect of TLR4 overexpression in sheep against invading pathogens are still not fully understood. In this study, using monocytes isolated from the peripheral blood of transgenic sheep, the expression of *TLR4* at the mRNA and protein levels was observed to be significantly higher than that in the WT monocytes. After *E. coli* infection, the mRNA level of *TLR4* increased in both groups but remained significantly higher in the TG group. The production of *TNFα* and *IL-6* along the TIRAP-MyD88-dependent pathway and the production of *IFN-β* by the TRAM-TRIF-dependent pathway were compared between the two groups, and the TG group showed significantly higher expression of these factors than the WT group. Since TLR4 is the only TLR that uses all four adaptors and activates both of these pathways [43], these two pathways are not activated simultaneously. Instead, the activation of these pathways is regulated by the location of TLR4. Higher activation of the TRAM-TRIF-dependent pathway indicates that more TLR4 is transferred from the cell surface to endosomes. Thus, the endocytosis pathway can activate the TRAM-TRIF-dependent pathway, which is illustrated by the TG group exhibiting higher levels of endocytosis than the WT group. This finding is consistent with other studies [11, 47, 48]. This inference was validated by experiments using monocytes infected with *E. coli* followed by CFU counts. However, there was no difference in Gram-positive bacteria. Inhibition of the TLR4 pathway significantly reduced the endocytosis and killing capacity of monocytes specific to the Gram-negative bacteria.

Endocytosis during the early stages of infection was further examined in the TG and WT monocytes using pHrodo- labelled *E. coli*. We found that while the percentage of monocyte endocytosis of pHrodo- labelled *E. coli* in the two groups increased with increasing infection time, the TG monocytes exhibited a higher percentage of endocytosis. Furthermore, the TLR4 inhibitor TAK242 reduced the percentage of internalization to a greater degree in the TG group. This finding may be attributable to TG monocytes having more TLR4 receptors on their surface, leading to more sensitive bacterial detection and more monocytes participating in bacterial internalization.

Cells have evolved a variety of strategies to endocytose particles, solutes, and bacteria [49]. TLR4 signalling is associated with receptor internalization and trafficking, and which endocytosis pathways are used during these processes is still not fully understood. The caveolae share a lipid composition with plasma membrane rafts and represent a subdomain that is stabilized by caveolin proteins. The formation of caveolae is highly complex and regulated by cellular processes [50]. It has been noted that rafts act as potential sites for LPS to interact with CD14, and TLR4, TLR4 and accessory proteins can associate with plasma membrane rafts. The TLR4-raft association is stimulated under LPS [48]. However, caveolae involvement in TLR4-mediated endocytosis has not been fully elucidated. Recent studies have shown that the deficiency of the protein LAPF (lysosome-associated and apoptosis-inducing protein containing pH and FYVE domains), which affects the TLR4 pathway, can impair the phagocytosis and killing of *E. coli* by influencing caveolae complex formation in macrophages of mice [21]. Here, by detecting the phosphorylation of key proteins, we aimed to determine whether the higher endocytosis and killing capacity of the TG monocytes was caused by increased levels of caveolae-mediated endocytosis. We found that the TG group exhibited earlier phosphorylation of Src and CAV1 following infection, and the levels of phosphorylated Src and CAV1 in the TG group were higher than those in the WT group at the same time points post-infection. In addition, Src is required for Src-CAV1 complex formation and bacterial endocytosis. The inhibition of Src and caveolae-mediated endocytosis reduced the endocytosis and bactericidal capacity of the TG group, which further indicated that caveolae-mediated endocytosis was implicated in the enhanced capacity of TG monocytes for antimicrobial defence.

Acidification and maturation of endosomes promote the killing of intracellular bacteria [51, 52]. However, whether TLR4 causes endosome acidification is controversial [16, 53]. We next explored the acidification and maturation of endosomes to examine the enhanced killing of intracellular bacteria by the TG group. The TG group showed increased acidification of the endosome, and the internalized bacteria showed greater colocalization with both the primary endosomal marker EEA1 and the lysosomal marker LAMP1 than those of the WT group. Inhibitors of Src, caveolae-mediated endocytosis, and TLR4 all reduced the degree of acidification in the TG group. Cathepsin B activity as an indicator of protein degradation ability was also examined. The activity of cathepsin B is strongly affected by pH, and increased pH leads to a reduction in cathepsin B activity [54]. Our results of cathepsin B activity showed the same trend as phagosome acidification. All the results may indicate that TLR4 and caveolae-mediated endocytosis regulate endosome acidification and maturation.

## Conclusions

Our results provide novel evidence of the underlying antibacterial mechanisms of TLR4 in the innate immunity of sheep. TLR4 overexpression in monocytes enhances their internalization and killing of bacteria via caveolae-dependent endocytosis and promotes their ability to fight against pathogens during the earlier stages of infection; these are the first results from a large animal model. In addition, for the first time, the pH of endosomes was detected by artificially controlling the expression of TLR4, and it was proven that in the case of exogenous stimuli, the increase in TLR4 expression was correlated with the decrease in endosomal pH. A significant decrease in pH can inhibit the growth of bacteria and reflects the maturation of endosomes. Further experimental results indicate that TLR4 and caveolae-mediated endocytosis regulate endosome acidification and maturation, and the overexpression of TLR4 can promote the maturation of endosomes and the killing of *E. coli* by enhancing the phosphorylation of Src and CAV1. Src and CAV1 can be used as potential targets to provide a theoretical basis for future breeding for disease resistance.

## Supplementary Information

**Additional file 1: Table S1.** Gene-specific primers for quantitative real-time polymerase chain reaction (qRT-PCR).

**Additional file 2: Figure S1.** (A) Bacterial internalization was detected in transgenic (TG) and wild-type (WT) monocytes after different periods of incubation with *Staphylococcus aureus* (MOI = 10). (B) *TLR4* expression of transgenic (TG) and wild-type (WT) monocytes before and after 30 min of *S. aureus* infection (MOI = 10).

**Additional file 3: Figure S2.** Flow cytometric analysis of the percentage of monocyte endocytosis of pHrodo- labelled *E. coli* after 10 min and 30 min of infection in the TG group and the WT group.

**Additional file 4: Figure S3.** Flow cytometric analysis of the effect on endocytosis of pHrodo- labelled *E. coli* in both groups of monocytes pretreated with TAK242.

**Additional file 5: Figure S4.** Flow cytometric analysis of the percentage of monocytes that endocytosed pHrodo- labelled *E. coli* (MOI = 10) in the TLR4-overexpressing cells pretreated with DMSO or the inhibitors dasatinib (3 μmol/L) or filipin (3 μmol/L) prior to 30 min of infection with *E. coli*.

## Data Availability

The data analyzed during the current study are available from the corresponding author on reasonable request.

## Abbreviations

TLR4: Toll-like receptor 4
*E. coli*: *Escherichia coli*
CAV1: Caveolin 1
TIR: Toll-interleukin1 receptor
TIRAP: Toll-interleukin 1 receptor domain containing adaptor protein
MyD88: Myeloid differentiation factor 88
TRAM: TRIF-related adaptor molecule
TRIF: TIR domain containing adaptor-inducing interferon-β
MD2: Myeloid differentiation protein 2
LPS: Lipopolysaccharide
MAPK: Mitogen-activated protein kinase
MAL: MyD88 adapter-like
PBS: Phosphate-buffered saline
FBS: Fetal bovine serum
FITC: Fluorescein isothiocyanate
CD14: Differentiation 14
DAPI: 4′,6-Diamidino-2-phenylindole
SDS: Sodium dodecyl sulfate
TBST: Tris-buffered saline containing 0.1% Tween 20
*S. aureus*: *Staphylococcus aureus*
LB: Luria-Bertani
OD: Optical density
qRT-PCR: Quantitative reverse-transcription polymerase chain reaction
CFU: Colony-forming unit
TG: Transgenic
WT: Wild-type
DMSO: Dimethyl sulfoxide
TNFα: Tumour necrosis factor α
IL-6: Interleukin 6
IFN-β: Interferon β
MOI: Multiplicity of infection
EEA1: Early endosome antigen 1
LAMP1: Lysosomal associated membrane protein 1
LAPF: Lysosome-associated and apoptosis-inducing protein containing PH and FYVE domains
