# Supplementary material for: Overexpression of Toll-like receptor 4 contributes to the internalization and elimination of Escherichia coli in sheep by enhancing caveolae-dependent endocytosis
Source: J Anim Sci Biotechnol. 2021 May 10;12:63. doi: 10.1186/s40104-021-00585-z (PMC8108469; doi:10.1186/s40104-021-00585-z)
Supplement: Supplementary file 1 — Additional file 1: Table S1. Gene-specific primers for quantitative real-time polymerase chain reaction (qRT-PCR). [file 40104_2021_585_MOESM1_ESM.docx]

**Supplementary table 1**

Gene-specific primers for quantitative real-time polymerase chain reaction (q RT-PCR).

| Gene Primer Accession number R^2^  Efficiency (%) |
| --- |
| *TLR4* F-ATCATCAGCGTGTCGGTTGTCA NM_001135930.1 0.9919 105.4  R-GCAGCCAGCAAGAAGCATCAG  *TNFα*  F-TCTACTCGCAGGTCCTCTTC NM_001024860.1  0.9998 103.4  R-TCGGCATAGTCCAGGTATTC |
| *IL6* F-CTGCTGGTCTTCTGGAGTATC  NM_001009392.1 0.9997 91.5  R-TGTGGCTGGAGTGGTTATTAG |
| *IFN-β*  F-AATCGTCTGGAGCCAATCTG  XM_004004400.4 0.9974 108  R-GATGTTCAGTCACGGAGGT |
| *GAPDH*  F-CTGACCTGCCGCCTGGAGAAA  NM_001190390.1 0.9953 99.8  R-GTAGAAGAGTGAGTGTCGCTGTT |

>NM_001135930.1 Ovis aries toll like receptor 4 (TLR4), mRNA

ATGATGGCGCGTGCCCGCCGGGCTGCGGCTCTGATCCCGGCCATGGCCATCCTCTCCTGCCTGAGAACCGAGAGCTGGGACCCTTGCGTACAGGTTGTTCCTAACATCAGTTACCAATGCATGGAGCTGAATCTCTACAAAATCCCCGACAACATCCCCATGTCAACCGAGATGCTGGACCTGAGCTTTAACTACCTGAGACATTTAGGCAGCCATAACTTCTCCAGGTTCCCAGAACTGCAAGTGCTGGATTTATCCAGATGCGAAATTAAGATTATTGAAGACAACACATTTCAGGGTCTAAACCACCTCTCCACCTTGATACTGACGGGAAACCCTATCCAGAGTTT

AGCCTGGGGAGCCTTTTCTGGGCTATCAAGTTTACAGAAGCTGGTGGCCGTGGAGACAAACCTAGTATCTCTAGATGACTTCCCCATTGGACATCTCAAAACCTTGAAAGAGCTTAATGTGGCTCACAATTTTATCCATTCCTTCAAGTTACCTGAATATTTTTCTAACCTTCCCAACCTGGAGCACTTGGATCTTTCTAACAACAAAATACAAAATATTTATTATGAAGATGTGAAAGTTCTACATCAAATGCCCCTACTCAACCTCTCTTTAGATTTGTCCCTGAACCCTTTAGAATTTATTGAACCAGGTACCTTTAAAGAAATTAAGCTCAATGGATTGACTCTGCGAAGTAATTTTAACAGTTCAGATGTCATGAAAACTTGTATTCAAGGTCTGGCTGGTTTAAAAATCAACCGGCTGGTTTTGGGAGAATTTAAAAATGAAAGGAAGTTGCAAAAATTTGACAGATCTTGCCTGGAGGGACTGTGCAACCTGACCATTGAGCAATTCCGGATAGCGTACTTGAGCAAATTCTCATGGAACGATACAGACTTATTTAATTGTTTGGCAAATGTTTCTGTGATTTCTCTGTTGAGTATATCTTTAGGAAGTCTACAAGCCCTTCTTAAAGATTTTAGATGGCAACACTTAGAAATGATTAACTGTGACTTTGATAAGTTTCCTGCACTGAAGCTCCGTTCTCTCAAAAAGTTTGTTTTCACAGACAACAAAGATGTAAGCACTTTTACTAAGACTGAGCTACCAAGCCTTCAGTATCTAGATCTCAAAAGAAATCACTTGAGTTTCAAGAGCTGCTGTTCTCACACTGATTTTGGGACAACCAACCTGAAGCATTTAGATCTGAGCTTCAATGACGTCATTACCTTAGGTTCAAACTTCATGGGCTTAGAGCAGCTAGAACACCTGGATTTTCAGCATTCCACTCTGAAACAGATCAATGCTTTTTCAACGTTCCTATCACTCAGAAACCTCCGCTACCTTGACATCTCTTACACCAACATCCGGATTGTCTTCCACGGCATCTTTACTGGCTTAGTCAGTCTGCAGACCTTGAAAATGGCAGGCAACTCTTTTCAGAACAACTTGCTCCCTGAC

ATCTTCACAGAGCTGACTAACTTAACCATCTTGGACCTCTCTAAGTGTCAACTGGAACAGGTGTCCTGGGCGGCATTTCACTCCCTCCCTAGCCTTCAGGTGCTGAATATGAGTCACAACAAACTCTTGTCATTGGATACATTTCTTTATGAACCACTCCACTCGCTCCGGATCCTAGACTGCAGTTTCAACCGTATCACGGCCTCTAAGGAGCAAGAACTACGGAATTTGCCAAGGAACCTCACTTGGCTAAATCTTACTCAGAATGAATTTGCTTGTGTTTGTGAACATCAGAGTTTCCTGCAGTGGGTCAAGGACCAGAGGCAGCTCTTGGTGGGAGCTGAGCAAATGATGTGTGCAGAGCCTTTAGATGTGAAGGACATGCCAGTGCTTGGTTTCAGGAATGCCACTTGTCAGATGAGCAAGACGATCATCAGCGTGTCGGTTGTCACTGTACTCCTGGTATCTGTGGTAGGAGTCCTAGTCTATAAGTTCTATTTCCACCTGATGCTTCTTGCTGGCTGCAAAAAGTATGGCAGAGGTGAAAGCACCTATGATGCCTTTGTGATCTACTCGAGCCAGGATGAAGCCTGGGTGCGGAATGAACTGGTAAAGAACTTGGAGGAGGGCGTGCCCCCCTTTCAGCTCTGCCTTCACTACAGGGACTTTATTCCTGGGGTGGCCATCGCCGCCAATATCA

TCCAGGAAGGTTTCCACAAGAGCCGTAAGGTGATTGTCGTGGTGTCCCAGCACTTCATCCAGAGCCGATGGTGTATCTTCGAGTATGAGATTGCCCAGACCTGGCAGTTTCTGAGCAGCCGTGCTGGCATCATCTTCATCGTCCTGCAGAAGCTGGAGAAGTCTCTCCTGCGGCAGCAGGTGGAGCTCTATCGCCTTCTGAACAGGAACACCTACCTGGAGTGGGAGGACAGTGTCCTGGGGCGGCATGTCTTCTGGAGAAGACTCAGAAAAGCCTTGCTGGCTGGTAAGCCCCGGAGTCCAGAAGGAACAGCAGATGCAGAGACCAACCCGCAAGAAGCGACCACCTCCACCTGAGGAGGAGAATCCCCTGATGCGCTCCTTGCCCAGATGGATGCAGGGTGTGTTCAGTTAACAAGTAAATGCCGC

>NM_001024860.1 Ovis aries tumor necrosis factor (TNF), mRNA

GGGACACCAGGGGACCAGCCAAGAGAGAGACAAGCAGCTGCAGAACCCCCTGGAGATAACCTCCCAGACAACACACCCCCGAGAGACAGCCAGGCAACTTGCTCTCTCATACACCCTGCCACAAGGCTCTCCTGTCTCCCGTCTGGACTTGGATCCTTCTGAAAAAGACACCATGAGCACCAAAAGCATGATCCGGGATGTGGAGCTGGCGGAGGAGGTGCTCTCCAACAAAGCAGGGGGCCCCCAGGGCTCCAGAAGTTGCTGGTGCCTCAGCCTCTTCTCCTTCCTCCTGGTTGCAGGAGCCACCACGCTCTTCTGCCTGCTGCACTTCGGGGTAATCGGCCCCCAGAGGGAAGAGCAGTCCCCAGCTGGCCCCTCCTTCAACAGGCCTCTGGTTCAGACACTCAGGTCATCTTCTCAAGCCTCAAATAACAAGCCGGTAGCCCACGTTGTAGCCAACATCAGCGCTCCGGGGCAGCTCCGATGGGGGGACTCGTATGCCAATGCCCTCATGGCCAACGGCGTGGAGCTGAAAGACAACCAGCTGGTGGTGCCCACTGACGGGCTTTACCTCATCTACTCGCAGGTCCTCTTCAGGGGCCACGGCTGCCCTTCCACCCCCTTGTTCCTCACCCACACCATCAGCCGCATTGCAGTCTCCTACCAGACCAAGGTCAACATCCTCTCTGCCATCAAGAGCCCTTGCCACAGGGAGACCCTAGAGGGGGCTGAGGCCAAGCCCTGGTACGAACCCATCTACCAGGGAGGGGTCTTCCAGCTGGAGAAGGGAGATCGCCTCAGTGCTGAGATCAACCTGCCGGAATACCTGGACTATGCCGAGTCTGGGCAGGTCTACTTTGGGATCATCGCCCTGTGAGGGCGCAGGACATGCATCCTCTCCCACCTCAGTTACCTTATTATTTACTCCTTCAGACCCTCCTCATCCCCTTCTGGTTTAGAAAGGGAATTAGGGGCTCAGGGCTGGGCTCCAAGCGTCCAACTTTAAACAGCTGCACTTAGAAATTAGGGATGTAGGGAAGTGAGGCCTGGACAACGGGCCACCAACCATCACCAAGGACTGGAACTGGAACTTCCAGAACTCCCTCGGGTCCACAAGTTTGGGTTCCCGGATGCAACCTGGGACACCCAGAATGCAAGGGCCAGGGTTCTTACCGGAATACTTCGCAACGTTCCTTGAGAAGATCTCACCTAGAACTTGACATGGGTGGGCTTCAACTCTCCCTTCCTGCCAGTGTTTCCAGATTCCCCTGAGGTGGGAAGCCCAGCCCCAACCCCACTGGGCCAACTCCCTCTGTTTATGTTTGCACTTATGATTATTTATTATTTATTTATTATTTATTTATTTACTAATGAATGTATTTATTCAGGAGGTCAAGGTGTCCTGGGAGACACAAACTAAGGGCTGCCTTGGCTCAGATGTGTTTTCTGTGAAAACGGAGCTGAACTGCAGGTTGCTCCCACCATGCCTCCTGGCCTTTGTGCCTCCTTTTGCTTATGTTTTTTAAAAAATATTTATGTGATCAAGTTGTCTAAATGATGCTGATTTGGTGACTGATTTGTCGCTACATCACTGAACCTCCGCTCCCCAGGGGAGTCATGCCTGTAACCGCCCTACTGGTCAGTGGCGAGAAATAAAGTGTCCTGAGAAAAGAAAAAAAAAAAAAAA

>NM_001009392.1 Ovis aries interleukin 6 (IL6), mRNA

CACCAGGAACGAAAGAGAGCTCCATCAGCCCTCCAGGAACCCAGCTATGAACTCCCTCTTCACAAGCGCCTTCAGTCCACTCGCTGTCTCCCTGGGGCTGCTCCTGGTGATGACTTCTGCTTTCCCTACCCCGGGTCCCCTGGGAGAAGATTTCAAAAATGACACCACCCCAAGCAGACTACTTCTGACCACTCCAGAAAAAACCGAAGCTCTCATTAAGCACATCGTCGACAAAATCTCTGCAATAAGAAAGGAGATATGTGAGAAGAATGACGAGTGTGAAAACAGCAAGGAGACACTGGCAGAAAATAAGCTGAAACTTCCAAAAATGGAGGAAAAAGATGGATGCTTCCAATCTGGGTTCAATCAGGCGATTTGCTTGATCAAAACCACTGCTGGTCTTCTGGAGTATCAGATATACCTGGACTTCCTCCAGAACGAGTTTGAGGGAAATCAGGAAACTGTCATGGAGTTGCAGAGCAGTATCAGAACACTGATCCAGATCCTGAAGGAAAAGATCGCAGGTCTAATAACCACTCCAGCCACACACACTGACATGCTGGAGAAGATGCAGTCCTCAAACGAGTGGGTAAAGAACGCAAAGGTTATCATCATCCTGAGAAGCCTTGAGAATTTCCTGCAGTTCAGCCTGAGAGCTATTCGGATGAAGTAGCTGCGGCTCCCATGATTGTGGTAGTTCCTGGGCATTCCCTCCTCTGGTCAGAAACCTGTCCACTGGGCACATAACTTATGTTGTTCTCTATGAAGAACTAAAAGTATGAGCGTTAGGACACTATTTTATCTTTAATTTATTGATATTTAAATATGTGGTTTTGAGTTAATTTATATACATGATAGGTATTTATATTTTTATGAAGTGCCACTTGAAATATTTTATGTATTTGGTTTGAAAAAGCAACGTAAAAATGGCTATGCGGCTTGAACGTCCTTATTGTTTTGGAGCCAAATCATTTCTTGAAATGTGTAGGCTTACCTCAAAAAATTTGCTAACTTATGCATATTTTTAAAGGCACATTTATATTGTATTTActTATGTTTAGGCTGTTTTTATAACAATAAACTTCTTTTTTAAAGAAAAAAAAAA

>XM_004004400.4 PREDICTED: Ovis aries interferon beta-2 (LOC101103623), mRNA

CCTGATTTCACCATGACCTACCGGTGCCTCCTCCAGATGGTTCTCCTGCTGTGTCTCTCCACCACAGCTCTTTCCAGGAGCTACAACTTGCTTCGATTCCAACAAAGGCGGAGCTCTGTGGTGTGTCAGAACCTCCTGTGGCAGTTACCTTCAACTCCTCAACATTGCCTCGAGTTCAGGATGGACTTCCAGATGCCTGAGGAGATGAAGCAAGCACAGCAGTTCCGGAAGGAAGATGCCGTATTGGTCATGTATGAGATGCTCCAGCACATCTTCCATATTCTCACCAGAGACTTCTCCAGCACTGGCTGGTCTGAGACCATCATTGAGCACCTCCTTGTGGAACTCTA

TGGGCAGATGAATCGTCTGGAGCCAATCTGGAAGGAAATAATGCAGAAGAAAAACTCCACTATGGGAGACACGACTGATCTTCACCTGCGGAAATATTACTTCAACCTCGTGCAGTACCTCAAGTCCAAGGAGTACAACAGGTGTGCCTGGACAGTCGTTCGAGTGCAAATCCTCAGGAACTTTTCTTTCCTGAGGAGACTAACAGGTTACCTCCGTGACTGAACATCTCCCCCCTGTGGCTCTGGGAAGGGACAATGTGACTTTGAGGTGAGACTCTTCAGCAGCAGAGGCTCTTG

>NM_001190390.1 Ovis aries glyceraldehyde-3-phosphate dehydrogenase (GAPDH), mRNA

GTAACTTCTGTGCTGTGCCAGCCGCATCCCTGAGACAAGATGGTGAAGGTCGGAGTGAACGGATTTGGCCGCATCGGGCGCCTGGTCACCAGGGCTGCTTTTAATACTGGCAAAGTGGACATCGTTGCCATCAATGACCCCTTCATTGACCTTCACTACATGGTCTACATGTTCCAGTATGATTCCACCCATGGCAAGTTCCACGGCACAGTCAAGGCAGAGAACGGGAAGCTCGTCATCAATGGAAAGGCCATCACCATCTTCCAGGAGCGAGATCCTGCCAACATCAAGTGGGGTGATGCTGGTGCTGAGTACGTGGTGGAGTCCACTGGGGTCTTCACTACCATGGAGAAGGCTGGGGCTCACCTGAAGGGTGGCGCCAAGAGGGTCATCATCTCTGCACCTTCTGCTGACGCTCCCATGTTTGTGATGGGCGTGAACCACGAGAAGTATAACAATACCCTCAAGATTGTCAGCAATGCCTCCTGCACCACCAACTGCTTGGCCCCCCTGGCCAAGGTCATCCATGACCACTTTGGCATCGTGGAGGGACTTATGACCACTGTCCACGCCATCACTGCCACCCAGAAGACTGTGGATGGCCCTTCCGGGAAGCTGTGGCGTGATGGCCGAGGGGCTGCCCAGAACATCATCCCTGCTTCTACTGGCGCTGCCAAGGCCGTGGGCAAGGTCATCCCTGAGCTCAACGGGAAGCTCACTGGCATGGCCTTCCGCGTCCCCACCCCCAACGTGTCCGTTGTGGATCTGACCTGCCGCCTGGAGAAACCTGCCAAGTATGATGAGATCAAGAAGGTGGTGAAGCAGGCCTCAGAGGGCCCTCTCAAGGGCATTCTAGGCTACACTGAGGACCAGGTTGTCTCCTGCGACTTCAACAGCGACACTCACTCTTCTACCTTCGATGCTGGGGCTGGCATTGCCCTCAACGACCACTTTGTCAAGCTCATTTCCTGGTACGACAATGAATTCGGCTACAGCAACAGGGTGGTGGACCTCATGGTCCACATGGCCTCCAAGGAGTAAGGTCCTTGGACCCCCAGCCCCAGCAGGAGCACGAGAGGAAGAGAGAGTTCCTCAGCTGCTGGGGAGTCCTGCCCCACCTCCACCACACTGAGAATCTCCCGACCTCCATACATTTCCATCCTCAAGGCCCTGAGGAAAGGGAGGGGCTTAGGGAGCCCTGCCTTGTCACGTACCATCAATAAAAGTACCCTATACCCAGAAAAAAAAAAAAAAAAAAAAAAAAAAAAAAAAAAAAAAAAAAAAA

Justification for the utility of GAPDH as a housekeeping gene in this experiment

The program geNorm [1] provides a measure of gene expression stability. The stability values calculated by geNorm were used to rank gene expression in reference genes. Seven candidate housekeeping genes were involved in the experiment, the *GAPDH* performed best in geNorm. In addition, we analyzed the *GAPDH* Cq value of each group, and there was no significant change between each group. Our results provided that *GAPDH* was stable after treatment and could be used as a housekeeping gene in our experiment.

We extracted RNA from four groups of monocytes, including two untreated groups (WT, TG) and two groups treated with *E. coli* for 30 min (WT, TG). First cDNA was synthesized from 1 ug of total RNA from each sample. Then, seven genes were selected from commonly used reference genes [2]. The primers for *GAPDH* are used as mentioned in method. Primers for *YWHAZ,* *RPL19,* *G6PD*, *SDHA,* *B2M* and *ACTB* were obtained from the references [3]. *YWHAZ* forward (F), 5′- AGACGGAAGGTGCTGAGAAA-3′ and reverse (R), 5′- CGTTGGGGATCAAGAACTTT-3′, *RPL19* forward (F), 5′- AGCCTGTGACTGTCCATTCC-3′ and reverse (R), 5′-ACGTTACCTTCTCGGG CATT-3, *G6PD,* forward (F), 5′- TGACCTATGGCAACCGATACAA-3′ and reverse (R), 5′- CCGCAAAAGACATCCAGGAT-3, *SDHA*, forward (F), 5′- CATCCACTACATGACGGAGCA-3′ and reverse (R), 5′-ATCTTGCCATCTTCAGTTCTGCT-3, *B2M*, forward (F), 5′- CTGTC GCTGTCTGGACTGG-3′ and reverse (R), 5′-TTTGGCTTTCCATCTTCTGG-3, *ACTB*, forward (F), 5′- AGATGTGGATCAGCAAGCAG-3′ and reverse (R), 5′-CCAATCTCATCTCGTTTTCTG-3′.


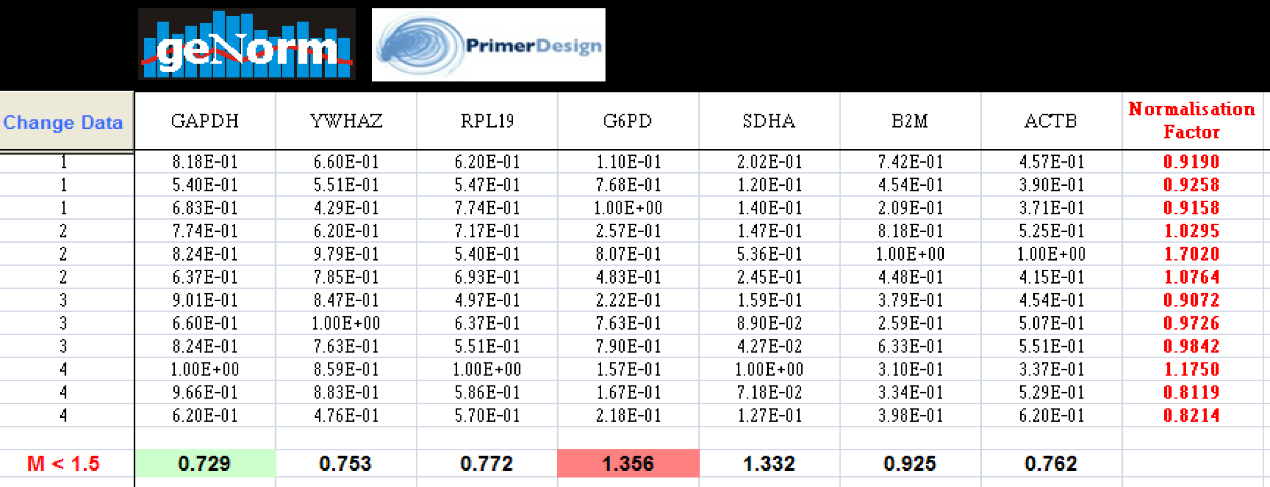


GeNorm analysis of reference genes.


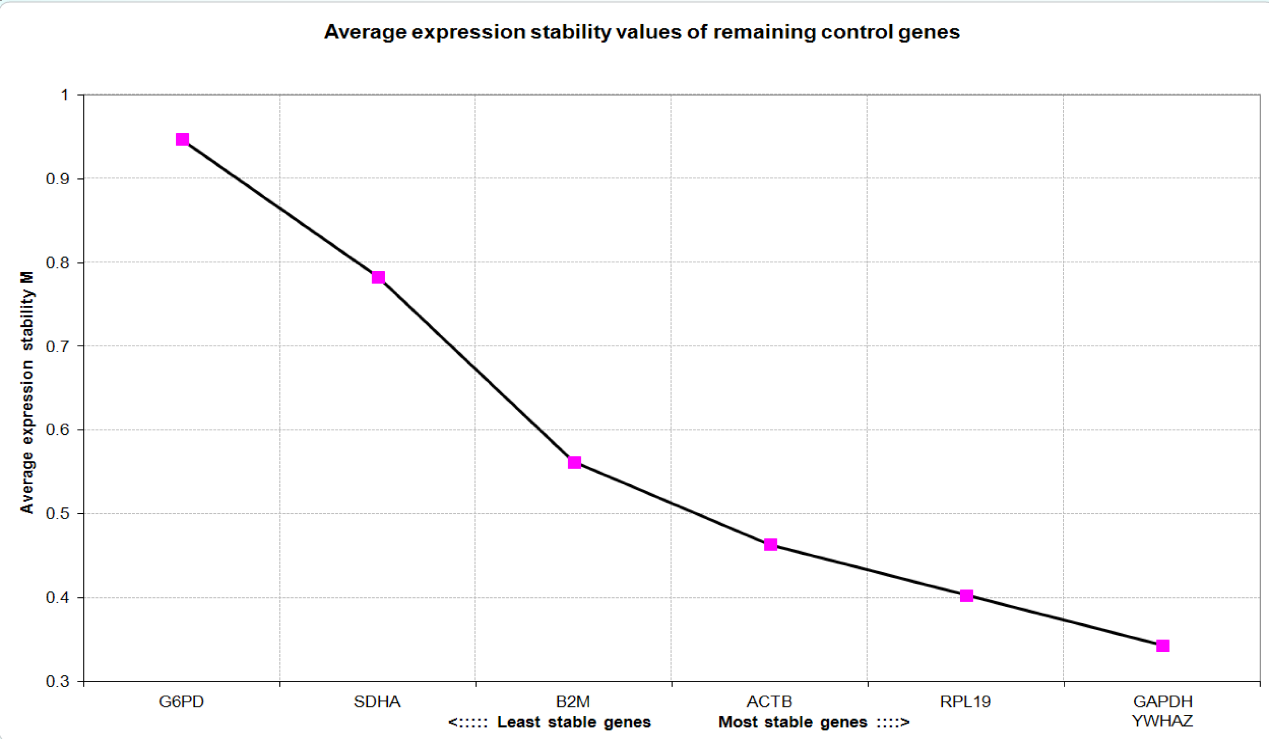


Stability ranking of candidate reference genes in Monocyte under *E. coli* treatment or not by the geNorm algorithm (lower stability values indicate more stable gene expression).


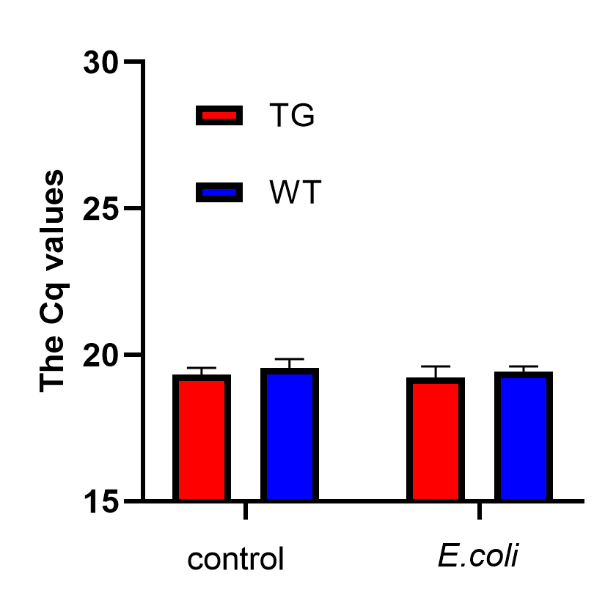


Cq values of *GAPDH* in each group including WT and TG untreated groups, WT and TG treated with *E. coli* for 30 min.

[1] Warrington JA, Nair A, Mahadevappa M, Tsyganskaya M. Comparison of human adult and fetal expression and identification of 535 housekeeping/maintenance genes. Physiol Genomics. 2000;2(3): 143-7. doi:10.1152/physiolgenomics.2000.2.3.143.

[2] Peletto S, Bertuzzi S, Campanella C, Modesto P, Maniaci MG, Bellino C et al. Evaluation of internal reference genes for quantitative expression analysis by real-time PCR in ovine whole blood. Int J Mol Sci. 2011;12(11): 7732-47. doi:10.3390/ijms12117732.

[3] Vorachek WR, Hugejiletu, Bobe G, Hall JA. Reference gene selection for quantitative PCR studies in sheep neutrophils. Int J Mol Sci. 2013;14(6): 11484-95.
