# Supplementary figures and images for: Overexpression of Toll-like receptor 4 contributes to the internalization and elimination of Escherichia coli in sheep by enhancing caveolae-dependent endocytosis
Source: J Anim Sci Biotechnol. 2021 May 10;12:63. doi: 10.1186/s40104-021-00585-z (PMC8108469; doi:10.1186/s40104-021-00585-z)

A

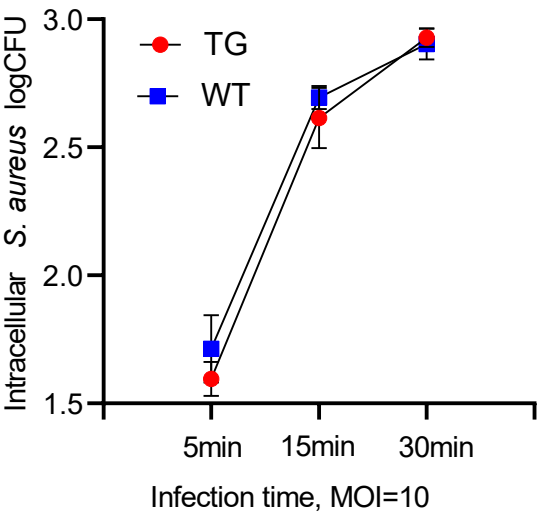

B

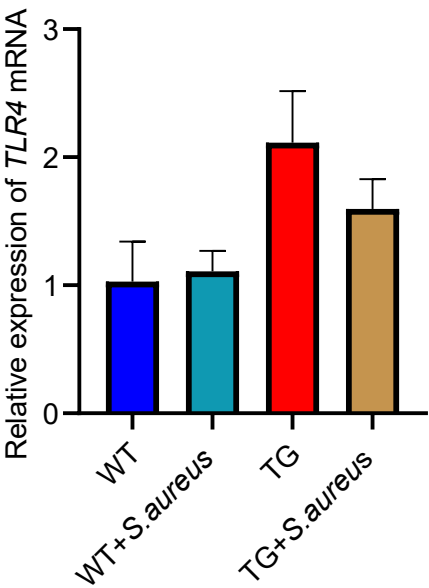

Supplement: Supplementary file 2 — Additional file 2: Figure S1. (A) Bacterial internalization was detected in transgenic (TG) and wild-type (WT) monocytes after different periods of incubation with Staphylococcus aureus (MOI = 10). (B) TLR4 expression of transgenic (TG) and wild-type (WT) monocytes before and after 30 min of S. aureus infection (MOI = 10). [file 40104_2021_585_MOESM2_ESM.pdf]

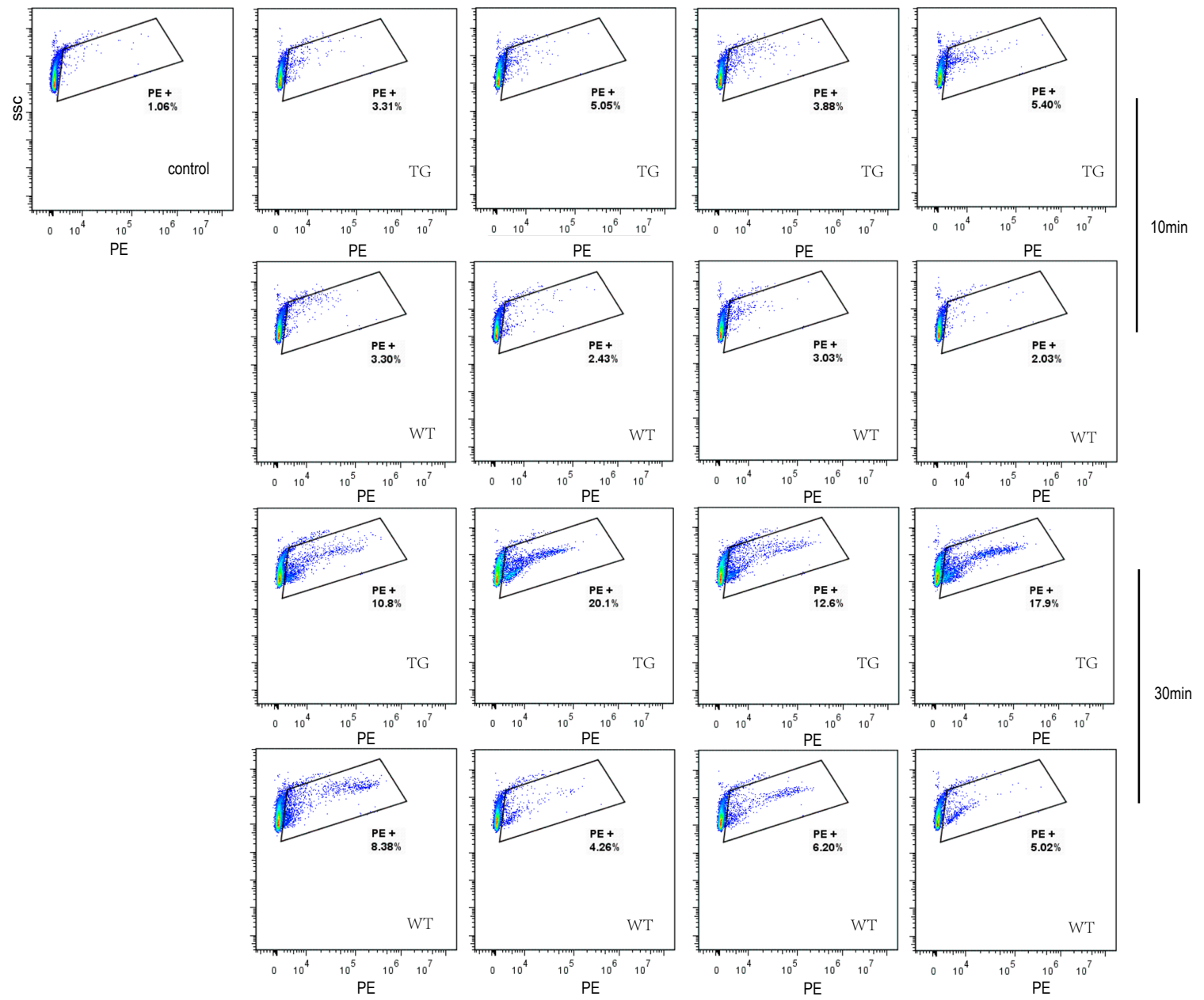

Supplement: Supplementary file 3 — Additional file 3: Figure S2. Flow cytometric analysis of the percentage of monocyte endocytosis of pHrodo- labelled E. coli after 10 min and 30 min of infection in the TG group and the WT group. [file 40104_2021_585_MOESM3_ESM.pdf]

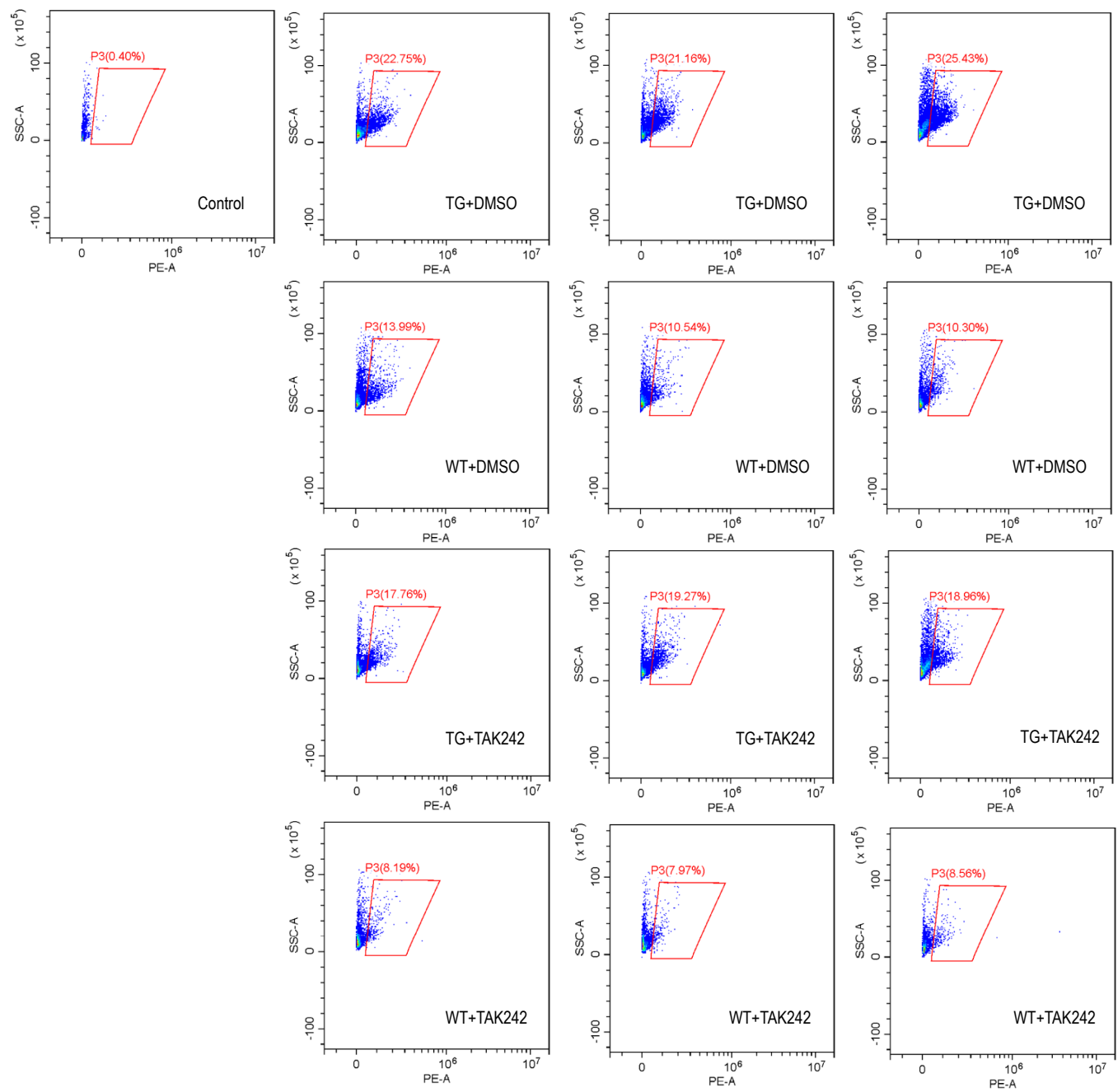

Supplement: Supplementary file 4 — Additional file 4: Figure S3. Flow cytometric analysis of the effect on endocytosis of pHrodo- labelled E. coli in both groups of monocytes pretreated with TAK242. [file 40104_2021_585_MOESM4_ESM.pdf]

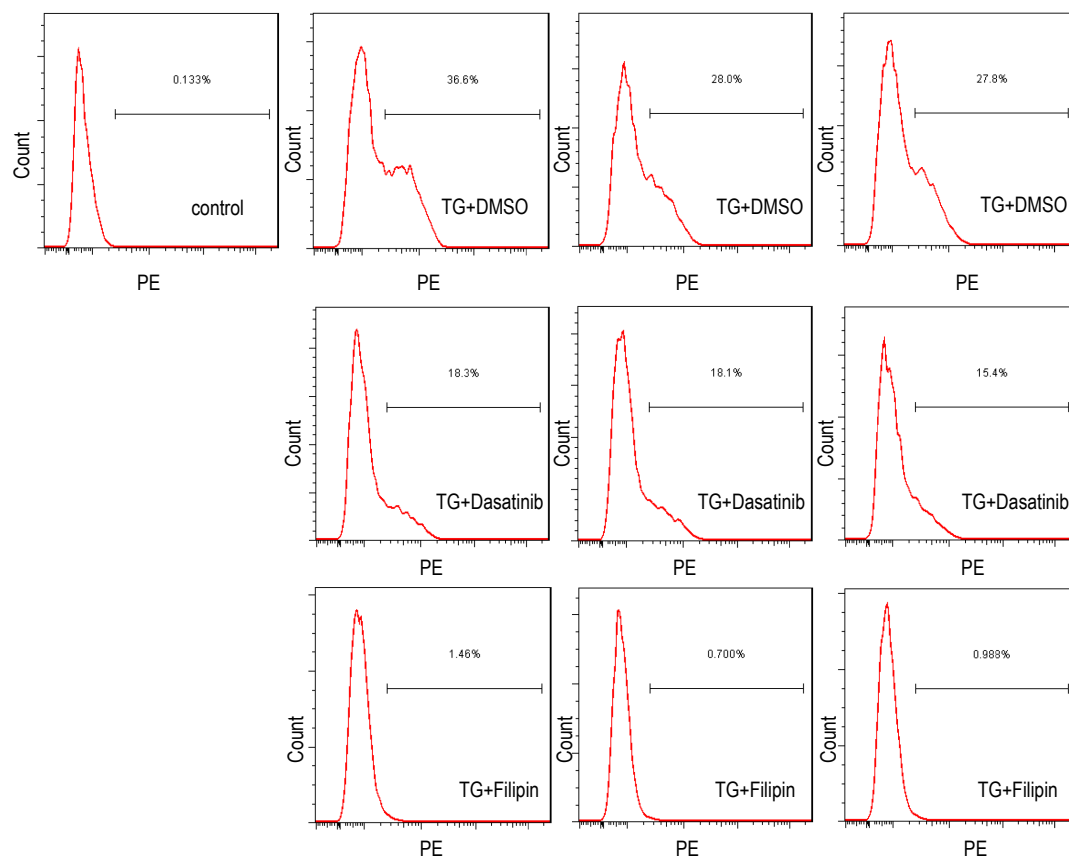

Supplement: Supplementary file 5 — Additional file 5: Figure S4. Flow cytometric analysis of the percentage of monocytes that endocytosed pHrodo- labelled E. coli (MOI = 10) in the TLR4-overexpressing cells pretreated with DMSO or the inhibitors dasatinib (3 μmol/L) or filipin (3 μmol/L) prior to 30 min of infection with E. coli. [file 40104_2021_585_MOESM5_ESM.pdf]
